# A nationwide survey of hospital-based thalassemia patients and standards of care and a preliminary assessment of the national prevention program in Sri Lanka

**DOI:** 10.1371/journal.pone.0220852

**Published:** 2019-08-16

**Authors:** Anuja P. Premawardhana, Rasnayaka Mudiyanse, Shamila T. De Silva, Nilam Jiffry, Udaya Nelumdeniya, Udaya de Silva, Sanath P. Lamabadusuriya, K. Pushpakumara, Randima Dissanayaka, M. Jansz, I. Rifaya, Upul Navarathne, V. Thirukumaran, Mahinda Arambepola, Wijesundara Dayanada Bandara, U. Vaidyanatha, Devan Mendis, K. Weerasekara, Nalika De Silva, D. K. Shantha Kumara, Sujeewa D. Amarasena, K. K. Hemantha, M. A. C. M. Refai, Ishari Silva, Nizri Hameed, F. Rajiyah, Sachith Mettananda, Angela Allen, David J. Weatherall, Nancy F. Oliveri

**Affiliations:** 1 Department of Medicine, Faculty of Medicine, University of Kelaniya, Sri Lanka; 2 Department of Pediatrics, Faculty of Medicine, University of Peradeniya, Sri Lanka; 3 Teaching Hospital, Kurunegala, Sri Lanka; 4 Provincial General Hospital, Badulla, Sri Lanka; 5 Teaching Hospital, Anuradhapura, Sri Lanka; 6 District General Hospital, Polonnaruwa, Sri Lanka; 7 General Hospital, Chilaw, Sri Lanka; 8 General Hospital, Vavuniya, Sri Lanka; 9 District General Hospital, Ampara, Sri Lanka; 10 District General Hospital, Monaragala, Sri Lanka; 11 Teaching Hospital, Batticaloa, Sri Lanka; 12 Teaching Hospital, Kandy, Sri Lanka; 13 District General Hospital, Matale, Sri Lanka; 14 Lady Ridgeway Hospital, Colombo, Sri Lanka; 15 District General Hospital, Matara, Sri Lanka; 16 Teaching Hospital Karapitiya, Galle, Sri Lanka; 17 District General Hospital, Hambantota, Sri Lanka; 18 Anti-Tuberculosis Campaign, Ministry of Health, Colombo, Sri Lanka; 19 Hemal's Adolescent and Adult Thalassemia Care Centre, North Colombo Teaching Hospital, Ragama, Sri Lanka; 20 Department of Pediatrics, Faculty of Medicine, University of Kelaniya, Sri Lanka; 21 Weatherall Institute of Molecular Medicine, University of Oxford, Oxford, United Kingdom; 22 University of Toronto, Toronto, Canada; University of Colombo Faculty of Medicine, SRI LANKA

## Abstract

**Objectives:**

Our aim was to describe the numbers and distribution of patients with different types of thalassemia and to assess the standards of care in all thalassemia treatment centers throughout Sri Lanka and the success of the ongoing prevention programme.

**Methods:**

This cross-sectional island-wide survey was conducted by two trained medical graduates, who visited each thalassemia center to collect data from every patient, using a standardized form. Data was collected through review of patient registers and clinical records.

**Results:**

We collected data on 1774 patients from 23 centers. 1219 patients (68.7%) had homozygous β-thalassemia, 360 patients (20.3%) had hemoglobin E β-thalassemia, and 50 patients (2%) had sickle β-thalassemia. There were unacceptably high serum ferritin levels in almost all centers. The annual number of births of patients with β-thalassaemia varied between 45–55, with little evidence of reduction over 19 years.

**Conclusions:**

Central coordination of the treatment and ultimately prevention of thalassemia is urgently needed in Sri Lanka. Development of expert centers with designated staff with sufficient resources will improve the quality of care and is preferred to managing patients in multiple small units.

## Introduction

The inherited disorders of hemoglobin are the most common monogenic diseases worldwide [1]. Approximately 300,000 babies affected with serious hemoglobin disorders, thalassemia and sickle cell disease are born annually [2]. While sickle cell disease syndromes are common in many African countries, thalassemia represents the most serious inherited disease and a major health burden throughout Asia. Patients with the most serious form, thalassemia major, require regular transfusions; most of the once-common disease complications arising from transfusional iron loading are preventable if early and adequate control of body iron is achieved with iron-chelating therapy [3], but many patients in lower-resource settings still sustain potentially fatal complications due to inadequate treatment [4].

Furthermore, although in 2006 the WHO designated thalassemia as a major public health concern [5] accurate information on the health burden arising from thalassemia in many Asian countries remains obscure. There is a concerning paucity of information with respect to patient numbers, genotypes, treatment requirements, disease-related complications and mortality. As a result, providing advice to governments and policy makers about the current or projected disease burden has been a daunting challenge [1].

In high resource countries over the last four decades, profound changes in the survival of patients with transfusion dependent thalassemia have been related to the improved safety of blood transfusions and to the long-term effectiveness of iron-chelating agents, including deferasirox, the only orally-active drug licensed for first-line treatment and prevention of iron loading. Heightened attention to complications including infection and, although generally limited to higher-resource countries, bone marrow transplantation, has also changed the prognosis for selected patients with thalassemia [4]. While most approaches to the pharmacologic augmentation of fetal hemoglobin in thalassemia have yielded modest effectiveness, some novel pharmacological and genetic-based therapies have shown early promise [6]. Such future approaches, while exciting, remain of limited relevance to many countries in Asia, where facilities for the diagnosis or management of the thalassemias remain extremely limited.

In any high prevalence country, successful treatment programs for thalassemia require an accurate understanding of the burden of disease, the concentration of patient numbers and the access to and limits on resources, particularly transfusion and effective first line iron-chelating therapy [1]. In high resource countries, adequately managed patients with thalassemia enjoy extended survival free of iron-induced complications and dramatically improved quality of life [3,7,8]. However, as is clear from many reports over the last decade, a disappointing high prevalence of complications is sustained in thalassemia patients throughout Asia and the Middle East [9,10,11,12,13].

The age distribution of thalassemia patients within a given country is also of critical importance. There is significant association in thalassemia between age and disease complications, with increasing incidences of iron-associated diabetes and cardiac, liver and other endocrine dysfunction in older, under-treated patients. Such complications, expensive and complicated to manage once established, create a requirement for additional medical resources over decades, but are largely preventable with early, effective therapy [3].

Adequate treatment programs should be applied ideally in parallel with effective programs of awareness and, where possible, prevention of new births. While many countries achieved success in the treatment of thalassemia at or around the same time that preventive approaches were introduced in the 1970s and 1980s [14], it is possible to achieve control of thalassemia by decreasing the number of new births through other approaches, particularly in relatively confined population such as in Sri Lanka. However, to encourage governments to promote these initiatives, it is important to have accurate data on the disease burden of thalassemia, not always a straightforward task: in our previous studies in this relatively small island there was a marked heterogeneity in the regional frequency of different forms of thalassemia [15].

Sri Lanka is a low middle-income country with a population of approximately 22 million; 75% of the population is of Sinhalese origin, 15% are Tamils and 9% are Moors [16]. The infant mortality rate of 8.4 per 1000 live births and maternal mortality ratio of 30 per 100,000 live births in 2017 [17] are among the lowest in the South Asian region [18]. Similarly, Sri Lanka has achieved remarkable success in preventive health, with the elimination of poliomyelitis in 2014, malaria and filariasis in 2016 [16], and 99% vaccination coverage for measles, mumps and rubella in 2017 [17]. These improvements in public health have resulted in a decline in mortality of children under the age of 5 years; as a result, babies born with serious genetic diseases, who would otherwise have died in infancy, survive to require diagnosis and management [18].

Thalassaemia is the commonest monogenic disease in Sri Lanka [15]. With public health resources exceeding those of many neighboring countries, Sri Lanka is well-positioned to improve management of its thalassemia population. Presently, while medical care and essential medicines for thalassemia are provided free of charge, there is no national registry or record of patient numbers or distribution. Country wide, adults may receive expert care from the dedicated adult thalassemia team at the North Colombo (Teaching) Hospital, Ragama. For others, as in most countries in Asia, the bulk of clinical management is implemented by pediatricians, general physicians and hematologists, all of whom would benefit from dedicated programs of training in the clinical management of thalassemia [19].

Previously in Sri Lanka, data on patient numbers and genotypes of thalassaemia was derived from estimates based on population surveys which used micro-mapping: in 2000, de Silva et al predicted a total patient number of approximately 2000, with one-third affected by Hemoglobin E β-thalassemia [15]. Subsequent information, obtained through self-reporting surveys in 19 centers, recorded 1547 patients with data available in 1379 patients [20]. That this was significantly less than predicted suggested the need for more organized efforts, to not only accurately estimate patient numbers and distribution, but also to define the burden of disease including its complications, as a potential impetus to the government, despite its limited resources, to prioritize thalassemia and improve patient care.

Since 2005/2006 a national program to screen individuals with the goal of reducing new births of children with thalassemia has been in place. This has involved the identification of carriers of thalassemia and measures to increase national awareness of thalassemia. There is no information to date on how or whether these efforts have impacted on numbers of births of patients with thalassemia.

One of the most fundamental issues with the thalassemia screening program is that it has operated without central control: individual hospitals conduct their own screening programs not collated by the Ministry of Health, in contrast to dengue or tuberculosis. Primarily, screening campaigns are conducted by the individual centers with visits to schools, factories, technical colleges and universities in parallel with public education programs. Another issue is that hemoglobin E trait and Hb S trait cannot confidently be excluded in any patient, because the process of screening involves the use of red cell size. In 2017, as an attempt to re-structure the programme, the process of screening using red cell size was continued but a new strategy of issuing advice to people with normal red cell indices (in whom hemoglobin E and S trait may have been missed) to obtain confirmatory High Performance Liquid Chromatography/ Capillary Electrophoresis of at least one partner at marriage, was implemented.

No national database on screening positives or "negatives” is available at present. The present system of follow-up of individuals at-risk does not permit claims to be made about the effectiveness of the program. Although documentation of births of patients with thalassemia major is admittedly incomplete, annual births showed no clear reduction after 2007.

The goal of this study was to define the numbers, genotypes, and distribution of patients with thalassemia throughout Sri Lanka. The study also aimed to identify the quality and variability of treatment available throughout the island, including the prevalence of complications and causes of death, to provide information about the adequacy of care of the country’s most common genetic disease. A secondary goal was to determine if information could be obtained on annual births of patients with thalassemia.

## Methods

All hospitals in the country with facilities for in-patient care were contacted by phone, to identify those managing patients with thalassemia. Twenty-six centers that manage patients with thalassaemia were identified and 25 centers consented to participate. Two centers were excluded due to logistical reasons and lack of manpower.

After permission was obtained from the administrative directors and consultants-in-charge of the thalassemia units at each hospital, two qualified medical graduates visited each center. Efforts were made to review all records of patients with thalassemia in each center; patient registers and clinical notes were accessed and, when available, patients were interviewed after obtaining informed written consent. Data were collected using a data collection form. Details about deaths were obtained from records, when available. Ethical approval was obtained from the Ethics Review Committee of the Faculty of Medicine, University of Kelaniya (P/185/09/2014).

## Results

**Fig 1:**
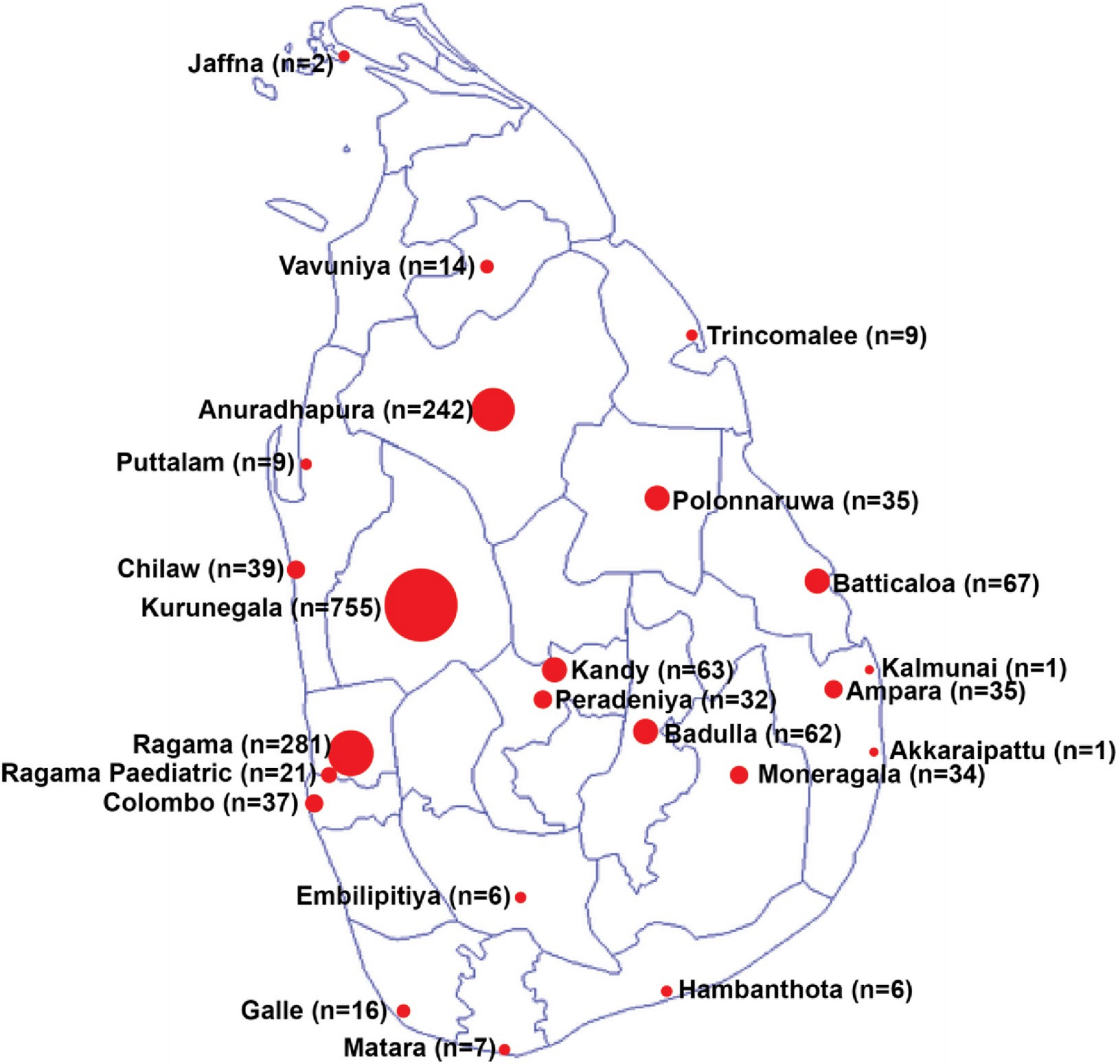
Distribution of centers with patient numbers.

In 23 centers 1774 patients with thalassemia were identified. Centers varied widely with respect to patient numbers: Six centers had more than 50 patients: Kurunegala (755), Ragama (281), Anuradhapura (242), Batticaloa (67), Kandy (63) and Badulla (62). Eight centers had less than 10 patients, and two centers (Akkaraipattu and Kalmunai) managed a single patient each. (Fig 1)

Most patients (1219; 68.7%) had been diagnosed with β-thalassemia major (defined as a requirement for ≥8 transfusions within a 12-month period); 360 patients (20.3%) had hemoglobin E (HbE) β-thalassemia and 89 (5.0%) had β-thalassemia intermedia (defined as a requirement for <8 transfusions within a 12-month period). Sixteen (0.9%) patients, although managed clinically as a major form of thalassemia, had been diagnosed as having β-thalassemia trait. In 29 patients (1.7%) the diagnosis was uncertain. There were no patients with the severe form of alpha thalassemia, haemoglobin H disease. (Table 1)

**Table 1:** Sex distribution of patients with different types of haemoglobinopathies.

*Clinical phenotype*	*Number*	*Male*		*Female*	
β-thalassemia major	1216	596	49.0%	620	51.0%
HbE β-thalassemia	360	167	46.4%	193	53.6%
β-thalassemia intermedia	89	44	49.4%	45	50.6%
Sickle β-thalassemia	50	26	52.0%	24	48.0%
δβ-thalassemia	7	4	57.1%	3	42.9%
β-thalassemia trait	16	6	37.5%	10	62.5%
Total		843	48.5%	895	51.5%

Patients with thalassemia major, with mean age of 13.2 (SD 7.6) years (range 5 months—44 years) were significantly younger than those with HbE β-thalassemia [mean age 21.5 (SD 12.9) years, range 6 months to 60 years; p<0.0001]. They were also significantly younger than those with thalassemia intermedia [mean age 33.6 (SD 18.8) years (range 4 months to 85 years); p<0.0001]. (Table 2)

**Table 2:** Age distribution of patients with different types of thalassaemia.

*Type of haemoglobinopathy*	*Total number of patients with recorded age*	*Mean (±SD) age (years)*	*Age range*
β-thalassemia major	1177	13.2 (±7.6)	5 months– 44 years
HbE β-thalassemia	341	21.5 (±12.9)	6 months– 60 years
β-thalassemia intermedia	83	33.6 (±18.8)	4 months– 85 years
Sickle β-thalassemia	46	20.1 (±12.7)	9 months– 47 years
δβ-thalassemia	7	29.3 (±15.5)	16–60 years
β-thalassemia trait	14	36.3 (±23.7)	5–72 years

Ethnically, 1450 patients were Sinhalese (at 82.6%, higher than the national representation at 72%), 211 were Moors (at 12.0%, higher than the national representation at 7.1%). Ninety-three were Tamils (at 5.3% less than one-third of the national representation at 18%) and 2 patients were Burgher (0.11%) [17].

### Births

**Fig 2:**
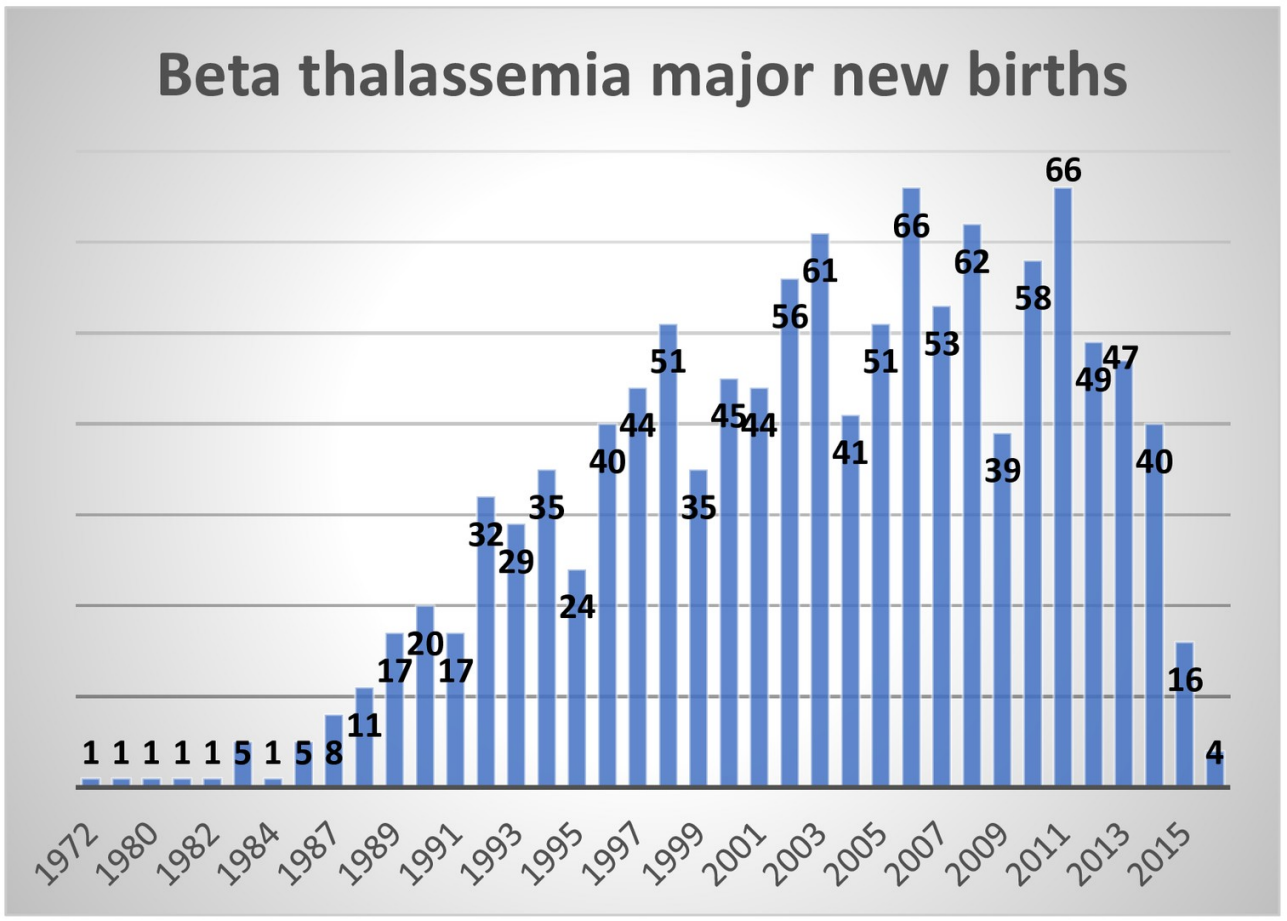
Beta thalassemia major new births from 1972 to 2015.

We also evaluated the number of births of patients with β-thalassaemia during a nineteen-year period from 1996 to 2014, by assessing birth records at each center. The number of new births, between 45–55 per year, had remained fairly constant during this period. (Fig 2)

### Patient number and quality of treatment received

There were six centers in which more than 50 patients (mean 245, SD 268.21) received care; in another eight centers, fewer than 10 patients (mean 5.125, SD 3.35) received care. There was no clear indication about how many support staff was provided to any center. Given the variation in clinic size, we anticipated there would be considerable variation in the management of patients at different centers. While many centers had a single pediatrician, lacking other support, some centers had ancillary health workers who assisted in clinical organization and care.

We used a surrogate marker, serum ferritin concentration, to estimate the adequacy of control of transfusional body iron burden, as one critical marker of the quality of treatment. Disappointingly, there was a paucity of reliable measurements of serum ferritin concentration in many centers. Overall, of 1774 patients, data on serum ferritin concentration was available in only 1131 (63.75%). Absence of data ranged between 0–100% in different centers. Overall, the mean value of serum ferritin concentration was 2382.7 ug/L (SD 2113.6). In 10 out of 18 (55.5%) centers in which serum ferritin measurements were available, mean ferritin exceeded the threshold 2500 ug/l for long-term survival free of clinical cardiac disease (Table 3).

**Table 3:** Mean serum ferritin value of patients in each center.

Centre	Number of Patients	Data available	Mean (±SD) serum ferritin values (mg/dL)
Badulla	62	41	2578 (±688)
Chilaw	39	19	1777 (±1484)
Akkaraipatttu	1	0	N/A
Ampara	35	30	3200 (±2018)
Anuradhapura	242	146	2604 (±1839)
Batticaloa	67	50	4014 (±2598)
Kalmunai	1	0	N/A
Peradeniya	32	19	2254 (±1617)
Polonnaruwa	35	14	2720 (±19560
Ragama	281	112	2022 (±1535)
Ragama Pediatric	21	18	1909 (±1091)
Trincomalee	9	0	N/A
Embilipitiya	6	3	9180 (±7320)
Galle	16	0	N/A
Hambantota	6	6	3617 (±2203)
Jaffna	2	0	N/A
Kandy	63	37	2639 (±2068)
Kurunegala	755	556	2099 (±2145)
Matara	7	4	1412 (±472)
Monaragala	34	22	4297 (±3178)
Puttalam	9	8	1746 (±1468)
Vavuniya	14	12	2018 (±1056)
LRH	37	21	2561 (±1891)

### Deaths

241 deaths were recorded and causes of death were not recorded in 177/241 (73.4%) of the available records. Records of deaths were available in only 7/23 (30.4%) centers. When recorded, heart failure accounted for 34/64 (53.1%) deaths, with infections also responsible for death in 25/64 (39%) of patients.

### Distribution of resources

Most adult patients were being managed in general pediatric wards, with only one center (Ragama) dedicated to the care of adult patients with thalassemia. Six centers managed more than 50 patients, whilst eight centers managed fewer than ten patients; three centers managed fewer than five patients. There were very few thaassaemia centres or patients in the Northern districts.

## Discussion

One of the most striking findings of our analysis was the young mean age (mean age of 13.3 years) of patients affected with thalassemia major, reflecting at least in part an unacceptably high incidence of premature death in this largest group of patients in the country. It is sobering that, as of 2017, Sri Lankan children with thalassemia achieve survivals similar to those reported in affected US children half a century ago [21]. This dismal prognosis contrasts sharply to current survivals in many North American centers of thalassemia patients up to age 60 years [7,8].

While this poor outcome reflects a long history of marginal treatment in Sri Lanka (prior to 1990 transfusions were irregularly administered and, prior to 1995 iron-chelating therapy was essentially unavailable), it also reflects twenty subsequent years of inadequate care throughout much of the country. Indeed, that deaths in children with thalassemia are still not considered particularly catastrophic is reflected in our identification of the inadequacy in most centers of records of such deaths: available in only 30% of centers, and those available documented cause(s) in only one in four deaths. While less regularly-transfused Sri Lankan patients, including those with hemoglobin E thalassemia, enjoy longer survivals, even their lives are prematurely shortened compared to comparable patients in higher-resource settings [22].

This premature mortality does not reflect lack of access to effective iron-chelating therapy: virtually all patients are treated with one of the two first-line agents, deferasirox or deferoxamine. Rather, this early deaths may be related to the overall quality of health care, which is a major driver of excess mortality worldwide [23]. A number of aspects of thalassemia care influence survival and are critical to programs of successful care. As in all chronic diseases, particularly those in which daily therapy is required, patient compliance, effective management of complications, quality of life, and survival are all improved by expert, dedicated, individualized attention, particularly for patients during the critical period of adolescence and young adulthood. Unfortunately, with only one specialized center for adult thalassemia care for the whole country, most adolescents and adults with thalassemia continue to be managed by pediatricians or by physicians with minimum training in the management of thalassemia. Our analysis identifies, in many patients, an unsuccessful struggle with therapy, as indicated by unacceptably elevated serum ferritin concentrations indicating insufficiently-controlled body iron burden.

In addition, record keeping was inadequate to provide information on common complications of iron loading including diabetes, hypothyroidism and liver dysfunction, which influence health, survival and quality of life, and are generally preventable with expert, dedicated management.

A second striking finding of our analysis was the apparently skewed ethnic distribution of patients. Previous population surveys have shown the prevalence of beta thalassemia to be almost equal in all three main ethnic groups, ie Sinhalese, Tamils and Moors [15,20]. However, our analysis identified severe thalassaemia to be less prevalent among ethnic Tamils, lower than among Tamils in South India. Tamil patients may have migrated during the recent ethnic conflict in the country.

Our previous survey identified widely variable distribution of thalassemia across short geographical distances [15]. This numbers of patients in different centers are consistent with these previous observations, except for the expected referral bias to the only dedicated center for adolescents and adults in Ragama. However, while patient distribution was consistent with our previous findings, patient numbers were not. Most patients with severe thalassemia (thalassemia major) were identified, but our analysis identified only about 50% of expected patients (based upon the previous analysis in 2000) with the generally milder form, hemoglobin E thalassemia. Our analysis also highlights a clear unequal allocation of resources. Six centers were managing over 50 patients, while three centers managed less than five patients.

Finally, in part because of the problems described above in the screening program initiated in 2007, it is impossible to determine, ten years after this program was implemented, whether there has been any impact on new births.

A number of conclusions may be drawn from our analysis. The most fundamental of these is that for all patients with thalassemia, expert care, with attention to judicious transfusion and appropriate chelating strategies, and expansion of that care to remote centers, are urgently needed in Sri Lanka. Consideration should therefore be given to several key areas.

First, centralized regional treatment centers staffed by and dedicated physicians, nurses, counselors and other support staff, expertly trained in the care and support of thalassemia patients should be set up. Each regional center should be supported sufficiently to absorb the care of patients now receiving care in smaller units. Such centralization of care, proposed fifty years ago in the United States for management of sickle cell disease [24], was successful in extending life expectancy in this disease. In all units, there is a critical need for physicians trained in the care of adult and adolescent patients with thalassaemia.

There is also a requirement for streamlined data entry and management including the implementation of an “at bedside” electronic record-keeping system at every center, with a central database maintained by collaborative experts.

The cost-benefit of a program to provide an alternative, curative approach to thalassemia, achieved through bone marrow transplanted from a compatible sibling donor, should be considered. Lifelong treatment costs following successful bone marrow transplant (BMT) are minimal, by contrast to those of transfusion and adequate chelation therapy. Several successful BMTs in Sri Lankan patients have already been carried out in a private sector program initiated in 2015, available only to richer patients [25]. A government sponsored BMT might be considered a cost-effective strategy as it has been in other high-risk countries, for the management of this disease.

The most common inherited disorder in Sri Lanka, thalassemia, does not receive focused attention in the undergraduate or postgraduate medical or paediatric curricula. Focused education in disease management, including with respect to management of patients with sickling disorders, needs to be expanded. Consideration should be given to the establishment of a national program of newborn screening for sickle cell disease.

Lastly, in parallel with improved care, the screening and awareness programs needs to be monitored by the Ministry of Health to achieve the desired primary outcome: reduction of affected births. Religious or legal authorities should sensitively monitor discussion in marriage counseling, since enforcing will not be ethical.

The gaps we have identified with respect to numbers, genotypes, complications, and laboratory evaluations indicate that thalassemia patients are not adequately “captured” by the country’s health care system. This is particularly troublesome given that Sri Lanka has, by contrast, well-organized national prevention and control programs in malaria, leprosy, tuberculosis, filaria and dengue. This inequity demands the urgent attention of policymakers, because, with minimal migration within the country and without any evident effect of the screening program in the reduction of new births, thalassemia patient numbers will increase in Sri Lanka over the next decade. We previously estimated that management of thalassemia might require about 5% of the country’s total health budget [15]. To avoid such substantial resource allocation, approaches to thalassemia care need to be re-evaluated, and resources assigned equitably. Paradoxically, focused investment in expert care will prevent debilitating and expensive complications and ultimately reduce long-term costs of management.

## Supporting information

S1 Table (XLSX)
